# Clinical effects of surgical and Gamma Knife treatments on hippocampal sclerosis-induced intractable epilepsy of children below age 10 years

**DOI:** 10.12669/pjms.294.3259

**Published:** 2013

**Authors:** Aiju Xiao, Tuanjie Wang, Yunjiao Tian, Li Xu, Shujun Li, Fenglian Zhu

**Affiliations:** 1 Aiju Xiao, The First Affiliated Hospital of Xinxiang Medical University, Weihui 453100, P. R. China.; 2 Tuanjie Wang, The First Affiliated Hospital of Xinxiang Medical University, Weihui 453100, P. R. China.; 3 Yunjiao Tian, The First Affiliated Hospital of Xinxiang Medical University, Weihui 453100, P. R. China.; 4 Li Xu, The First Affiliated Hospital of Xinxiang Medical University, Weihui 453100, P. R. China.; 5 Shujun Li, The First Affiliated Hospital of Xinxiang Medical University, Weihui 453100, P. R. China.; 6 Fenglian Zhu, The First Affiliated Hospital of Xinxiang Medical University, Weihui 453100, P. R. China.

**Keywords:** Hippocampus sclerosis, Intractable epilepsy, Surgery, Gamma Knife

## Abstract

***Objective:*** To discuss the treatment effects and costs of surgery and Gamma Knife on hippocampal sclerosis (HS)-induced intractable epilepsy of children below age 10 years.

***Methods:*** The children below age 10 years who suffered from HS-induced intractable epilepsy from June 2010 to June 2012 were subjected to surgical and Gamma Knife treatments respectively according to their preference.

***Results:*** The short-term curative rates of the surgical group and the Gamma Knife group were 93.51% and 54.87%, respectively. The average expenses of the two groups were 10,000 CNY (Chinese Yuan) and 22,000 CNY, respectively.

***Conclusion:*** The two groups were treated safely and effectively, but the surgical treatment led to better results at a reduced cost.

## INTRODUCTION

Intractable epilepsy, which is also known as refractory epilepsy, refers to the epilepsies that cannot be controlled by drugs. This may commonly result from hippocampal sclerosis (HS).^1^ This paper studied the treatment methods concerning HS-induced refractory epilepsy of children below age 10. Currently, surgical treatment has been widely applied in HS-induced intractable epilepsy,^2^ which is safe and effective.^3^ On the other hand, Gamma Knife has also been utilized in recent years.^4^ In this study, the children below age 10 years who suffered from HS-induced intractable epilepsy from June 2010 to June 2012 were subjected to surgical (154 cases) and Gamma Knife (82 cases) treatments respectively according to their preferences. The children were followed-up after completion of treatment. 

## METHODS

***Subjects:*** Two hundred thirty six children patients suffering from uncontrollable medial temporal lobe epilepsy (MTLE) after receiving formal anti-epileptic treatment in our hospital from June 2010 to June 2011 were selected as the subjects for the study. .

***Preoperative examinations:*** The children who had been examined by Video-EEG (V/EEG) and MRI were subjected to SPECT scan^5^ and diagnosed as HS-induced refractory epilepsy. Eighty two children were subjected to Gamma Knife treatment, and 154 children underwent craniotomy. Wada tests were also performed on all those who had lesions on the left and some with right sided lesions ^6^

***Evaluation criteria:***

*The patients were evaluated following the treatment in 5 outcome classes:*

*Class *I: No seizures in the past six months (the cases with auras were excluded). 

Class II: The frequency of seizure was lowered by at least 50% compared to the preoperative six months. 

Class III: The seizure was lowered by less than 50% compared to the preoperative six months. Class IV: Seizure were not significantly lowered. 

Class V: Seizures frequency was increased. 

The result of Classes I+II were considered an effective outcome.

***Grouping and case description: ***Surgical group (n=154) which included 99 male and 55 female. Age of disease onset<10 years old and Surgical age< 10 years old. The Gamma Knife group had 82 cases, 50 male and 32 female. Age of disease onset<10 years old and Surgical age< 10 years old.

Average follow-up time was 6-32 months (median: 10 months) in Surgical group and 12-54 months (median: 33 months) in Gamma Knife group. The follow-up results of the two groups did not differ significantly (p=0.0008).

## RESULTS

***Disease history and types:*** The disease history and types of the 2 groups are listed in Table-I.

**Table-I:** Disease history and types

*Item*		*Surgical (n=154)*	*Gamma Knife (n=82)*
History	Febrile convulsion	72	63
	Trauma	11	6
	Diarrhea and dehydration	6	1
	No particular	45	6
	Undefined	22	6
Symptomatic sign (Aura)	Chest discomfort	77	32
	Abdominal discomfort	18	25
	Tension	24	0
	Dizziness	11	6
	Imagination disorder	10	13
	None	14	6
Type	SPS	72	31
	CPS	154	82
	SGS	99	44
Disease course (year)	Range	2-10	3-10
	Median	4.6	5.3
HS side	Left	66	32
	Right	88	50

SPS = simple partial seizures CPS = Complex Partial Seizures SGS = Secondary Generalized Seizures

***Auxiliary examinations:*** All the 236 patients were subjected to intracranial MRI and interictal EEG tracings with sphenoid electrodes. The patients with epileptiform discharges spreading bilaterally and/or discordant MRI lesions also underwent V/EEG recording. The 16 patients in the surgery group with left HS, except for one case of hippocampal gliosis (MRI) and 5 cases of left-handed children, all underwent Wada test before the surgery by the carotid injection of 2-4 mg clonazepam.

***MRI and interictal EEG and V/EEG:*** The 236 children consisted of 98 cases of left HS and 138 cases of right HS. The interictal EEG of all patients, including tracings with sphenoid electrodes, were recorded. There were 132 cases of ipsilateral interictal epileptiform discharges, 88 cases of bilateral spread, and only five cases of contralateral MRI focuses, respectively. Patients who had interictal bilateral epileptiform discharges or discordant MRI lesions, had ictal EEGs recorded during onset at V/EEG. The results verify that the patients with contralateral interictal epileptiform discharges should be continuously monitored.

In the surgical treatment group, there were 12 patients without an ictal EEG. Of these seven patients had long-term EEG monitoring for 12 to 42h and at least two records of complete sleep cycles at night. During the entire record, the ratio of epileptiform discharges on the MRI lesion side to those on its contra-lateral side was greater than 93%. Three patients had only 2 to 14 interictal EEG records. As their clinical manifestations and MRI were typical CPS caused by HS, they received surgical treatment directly and all of them gained a short-term post-operative effect being completely free of seizures.

***Wada test:*** 39 patients passed the Wada test at one time (≥5/6), and three cases failed (≤4/6), demonstrating that the bilateral temporal lobes were involved in memory and language processes.

***Follow-up: ***The follow-up results are shown in Table-II.

**Table-II:** Follow-up results

*Item*		*Surgical (n=154)*	*Gamma Knife (n=82)*
Class of Seizure outcome	I	111 (72.08)	13 (15.85)
	II	33 (21.43)	32 (39.02)
	III	10 (6.49)	37 (45.12)
	Time to Termination of Seizure	Immediately after surgery (83 cases) Postoperative 3 months (39 cases)	16 months-2 years
Delayed edema		0	32
Average expense (CNY)		10000	22000

***Treatment effects:*** Both surgical lesion excision and Gamma Knife lesion irradiation were safe and effective treatment methods for HS-induced intractable epilepsy.^7^ However, the short-term effect of surgical treatment (93.51%) was better than that of Gamma Knife (54.87%) (P = 0.0015).^8^ Besides, the cost-effectiveness of the former was greater than that of the latter.^9^ The surgical therapy was effective immediately, while Gamma Knife treatment commonly reduced seizures a few years or several months later.^10^ No delayed cerebral edema occurred after the surgery. On the contrary, the patients treated by Gamma Knife treatment might suffer from symptoms several months after the treatment, which was often accompanied by cerebral edema requiring drug therapy, including corticosteroid and dehydrating agents. The occurrence of cerebral edema and the radiation dose were not significantly correlated.^11^

## DISCUSSION

This study involved numerous cases, thus rendering our conclusions representative and informative. In this study, the follow-up time of the two groups differed significantly. It has been previously reported that Gamma Knife usually cause beneficial effect mostly in postoperative 8-16 months^12^ rather than immediately.^13^ However, most studies have reported that anteromedial temporal resections reduced seizures immediately.^14^

HS-induced intractable epilepsy should be treated surgically as soon as possible, which has become an indisputable principle in the field of medical sciences^15^ that is equally safe and effective as appendectomy.^16^ 1) For the patients with refractory complex partial onset (with or without secondary generalized attack), if the treatment of the first-line anti-epileptic drug fails,^17^ the patients should be transferred to the epilepsy surgical treatment center.^18^ 2) If the center determines that the patients comply with the criteria of anteromedial temporal lobe resection, and the patients can tolerate with the surgical consequences and possible risks, we should treat them surgically instead of drugs.^19^ 3) The patients should not be recommended to receive only local neocortex resection owing to the lack of evidence.^20^ 4) The Gamma Knife group showed a significantly lower treatment effect than that of the surgery group and had postoperative seizure attacks recurrently for a long time.^21^ The patients in the Gamma Knife group suffered postoperative delayed cerebral edema, and the duration and time of occurrence were irregular, so the patients continued to suffer from headache and could not work normally. The difference in the effects between surgery and Gamma Knife treatments for medial temporal lobe epilepsy should be appreciated correctly,^22^ aiming to provide appropriate guidance for patients. The patients without surgical contraindications should be guided to receive surgical treatment.

There are also Dos and Don'ts in practical treatment: First, all the children enrolled in this study were in need of parents' guidance and consideration psychologically and physiologically. Secondly, the majority of patients in our country still live in poverty with the conditions of limited medical resources, we must treat the patients who can afford hospital services on the principles of "correct diagnosis, right guidance and high cost-effectiveness" to solve their problems reasonably and effectively.^23^ To better solve the above problems, the following aspects should be paid attention to: 1) To improve the understanding and diagnosis of MTLE, as well as the level to identify MTLE through EEG and MRI clinically is the first step to ensure patients to be correctly managed:^24^ A. In this study, we did not know that MTLE led to over 92% of the diseases. Therefore, it is of great significance to clarify MTLE symptomatologically. B. Inserting a sphenoid electrode for the EEG examination and understanding the diagnostic significance of EEG during the interictal period are crucial.^25^ C. Improving the level of MRI examination and introducing quantitative indicators are also critical.^26^ 2) Guiding patients appropriately to provide knowledge for them and their families concerning the safety and efficacy of MTLE. 3) The surgical treatment method is of greater cost-effectiveness compared to Gamma Knife.
